# High Prevalence of *Pantoea* spp. in Microbiota Associated with the Sorghum Plant Bug *Stenotus rubrovittatus* (Heteroptera: Miridae)

**DOI:** 10.1264/jsme2.ME22110

**Published:** 2023-07-13

**Authors:** Yuto Sato, Tatsuki Akao, Kazutaka Takeshita

**Affiliations:** 1 Faculty of Bioresource Sciences, Akita Prefectural University, 010–0195 Akita city, Japan

**Keywords:** sorghum plant bug, *Stenotus rubrovittatus*, *Pantoea*, insect-associated microbiota, 16S rRNA amplicon sequencing

## Abstract

The sorghum plant bug, *Stenotus rubrovittatus* (order Heteroptera: family Miridae), is a notorious insect pest in Japan that causes pecky rice. In the present study, we sampled this insect pest in the northern part of Honshu Island in Japan and investigated its associated microbiota. The results obtained showed that *Pantoea* dominated the associated microbiota and was the sole genus detected in all samples. The dominant *Pantoea* were phylogenetically close to rice pathogens. The present results suggest that the sorghum plant bug needs to be regarded and controlled not only as a notorious insect pest, but also as a potential vector of rice pathogenic *Pantoea* spp.

Insect-microbe symbiotic interactions are ubiquitous in nature (Buchner, 1965; Bourtzis and Miller, 2003; Douglas, 2015). Insect-associated microbes often affect the physiology and ecology of their hosts. In some cases, symbiotic microbes supply their host insects with essential nutrients for survival, such as essential amino acids and vitamins that cannot be obtained from the diet (Douglas, 2015), while in others, they change the host’s food availability and detoxify insecticides and phytotoxins (Hosokawa *et al.*, 2007; Itoh *et al.*, 2018; Sato *et al.*, 2021).

Rice is a major and important crop in Japan and Asia. The sorghum plant bug *Stenotus rubrovittatus* (order Heteroptera: family Miridae) (Fig. 1A) is a notorious insect pest in Japan that feeds on rice grains and causes pecky rice (Yasunaga *et al.*, 2001). This species has recently expanded its habitats from the southern to northern part of Japan, which includes famous regions for rice production, possibly due to global warming (Osawa *et al.*, 2018). Therefore, this insect pest has been attracting increasing attention in Japan (Tabuchi *et al.*, 2015).

In close relatives of the sorghum plant bug, *S. binotatus*, “*Rickettsia*-like symbionts” were found in the nuclei and cytoplasm of some epithelial cells of the digestive tract using transmission electronic microscopy (Chang and Musgrave, 1970). Microbial community ana­lyses were recently performed on some mirid bugs (Dally *et al.*, 2020; Luo *et al.*, 2021; Xue *et al.*, 2021). Despite the increasing attention on this insect pest, the associated microbiota of the sorghum plant bug remains unknown. Therefore, we herein investigated the microbiota associated with the sorghum plant bug *S. rubrovittatus* to obtain a more detailed understanding of the physiology and ecology of this insect pest. Refer to the Supplementary Materials and Methods for details on the materials and methods performed in the present study. The primers used in this study are listed in Supplementary Table S1.

Insect sampling was performed at 24 locations over seven prefectures (29 samples) in the northern part of Honshu Island in Japan (Supplementary Table S2). Between 0.11 and 5.7‍ ‍μg DNA (mean±SD, 2.2±0.15‍ ‍μg) was extracted from the whole insect (Supplementary Table S2). A clone library ana­lysis targeting a 1.5-kb fragment of the bacterial 16S rRNA gene was performed on the six samples collected in Akita city (three females and three males). Ninety-two clones were obtained and sequenced (15 or 16 clones from each sample) (Supplementary Table S2). These clones were classified into operational taxonomic units (OTUs) with vsearch 2.15.0 with a 99% identity threshold (Rognes *et al.*, 2016). Eleven OTUs were generated and subsequent homology searches of the represented clone sequences revealed that 5 of the 11 OTUs were affiliated with the genus *Pantoea* (Supplementary Table S3). The genus *Pantoea* (*Gammaproteobacteria: Enterobacteriaceae*) consists of versatile and diverse species and includes plant pathogenic species, clinical and environmental isolates, and symbiotic species of various insects, particularly pentatomid stink bugs (Walterson and Stavrinides, 2015; Duron and Noël, 2016; Hosokawa *et al.*, 2016, 2019; Itoh *et al.*, 2017; Lv *et al.*, 2022). Two of the five *Pantoea* OTUs were obtained from all six samples, independent of sex, and the five OTUs accounted for most clones (80 clones) (Supplementary Table S3).

We then attempted to isolate *Pantoea* bacteria from the digestive tract of the sorghum plant bug by plating the homogenate of each component of the digestive tract, namely, the midgut, first, second, and third sections, and hindgut (Supplementary Fig. S1). Emerging yellowish colonies, which is a characteristic of *Pantoea* spp. (Walterson and Stavrinides, 2015), were randomly selected and taxonomically identified by their 16S rRNA sequences. Seven *Pantoea* isolates were successfully cultured from all components of the digestive tract of the sorghum plant bug (Supplementary Table S4). The 16S rRNA sequences of the isolates suggested that these isolates were close relatives of the *Pantoea* OTUs detected in the clone library ana­lysis (Supplementary Table S3 and S4).

To understand the phylogenetic position of the five *Pantoea* OTUs and the seven *Pantoea* isolates derived from the sorghum plant bug, representative clone sequences from these OTUs, sequences of the isolates, those of representative *Pantoea* type strains, and those of *Pantoea* symbionts derived from other insects were subjected to a phylogenetic ana­lysis based on the maximum likelihood method (Fig. 1B). The five OTUs and seven isolates formed a monophyletic clade with several clones/isolates derived from other insects and several type strains, such as *P. ananatis* and *P. agglomerans*.

To examine the prevalence and relative abundance of *Pantoea* in wild populations of the sorghum plant bug, we performed amplicon sequencing targeting the V3–V4 region of the bacterial 16S rRNA gene with the next-generation sequencer for all 29 samples. Raw reads produced with Illumina Miseq (2×300 bp) were subjected to quality filtering, resulting in 16,501–63,999 (mean: 30,467) qualified and merged amplicon sequences per sample (Supplementary Table S2). These sequences were assigned to the genus level with RDP classifier 2.13 with an 80% confidence threshold (Wang *et al.*, 2007). The sequences assigned to Chloroplast were removed from subsequent ana­lyses. Consistent with the results of the clone library ana­lysis, in the six samples collected in Akita city, *Pantoea* sequences accounted for more than 60% of bacterial sequences (Fig. 2 and Supplementary Table S2). In all 29 samples, *Pantoea* was the sole genus accounting for more than 1%; however, the relative abundance of *Pantoea* sequences for the 29 samples varied at 1.4–95.7% (mean±SD, 56.1±29.9%) (Fig. 2 and Supplementary Table S2). “Unclassified_Enterobacterales” was detected in most samples; in samples with a low abundance of* Pantoea*, *e.g.*, OGT-F and NKH-F, genera, such as *Lactococcus*, *Staphylococcus*, and *Corynebacterium*, dominated (Fig. 2 and Supplementary Table S5). No sequences of *Rickettsia*, which had been indicated as symbionts in the mirid bug *S. binotatus* based on transmission electronic microscopic observations (Chang and Musgrave, 1970), were detected, except in one sample (Supplementary Table S5).

The size of the bacterial population associated with the sorghum plant bug was estimated by performing quantitative PCR targeting the bacterial 16S rRNA gene. The estimated size varied among samples from 2.9×10^3^ to 8.0×10^6^ (mean±SD, 8.3×10^5^±1.6×10^6^ gene copies insect^–1^) (Supplementary Table S2). Population sizes based on the number of bacterial 16S rRNA gene copies may have been overestimated because several bacteria had multiple copies in their genome, *e.g.*, *Pantoea* spp. were previously reported to have six copies (Stoddard *et al.*, 2015). A correlation was observed between the population size and relative abundance of *Pantoea* (Pearson’s correlation coefficient r=0.456).

The results of our microbial community ana­lyses showed that *Pantoea* dominated the associated microbiota and was the sole genus detected in all samples. Previous studies reported that some pentatomid stink bugs, *e.g.*, the brown-winged green stink bug *Plautia stali* and the sloe bug *Dolycoris baccarum*, develop midgut crypts, the lumen of which is colonized by *Pantoea* symbionts to establish obligate symbiosis (Hosokawa *et al.*, 2016; Itoh *et al.*, 2017). Some of these *Pantoea* symbionts formed a monophyletic clade with the *Pantoea* clones and isolates derived from the sorghum plant bug (Fig. 1B). The dominance and high prevalence in microbial community ana­lyses and the monophyletic relationship of the clones and isolates with *Pantoea* symbionts of the pentatomid stink bug suggest that the sorghum plant bug *S. rubrovittatus* also establishes a symbiotic interaction with *Pantoea* bacteria. However, the sorghum plant bug lacks midgut crypts, similar to many other mirid bugs (Supplementary Fig. S1) (Yanai and Iga, 1956; Chang and Musgrave, 1970). Although the successful culturing of *Pantoea* from other components of the digestive tract demonstrated their presence in the digestive tract, it is still possible that they were just transient via feeding. Their primary habitat may not be the digestive tract, but the body surface or salivary glands. In addition, mirid bugs are more likely to have a complex and unstable microbiota rather than established symbiotic interactions with a specific partner, such as the pentatomid stink bugs (present study; Dally *et al.*, 2020; Luo *et al.*, 2021; Xue *et al.*, 2021). Therefore, since we cannot conclude that *Pantoea* is a stably colonized symbiont of the sorghum plant bug, further investigations are needed on this relationship. In future studies, we will examine the localization of *Pantoea* inside the host body using fluorescence *in situ* hybridization and perform rearing experiments on the host insect with and without *Pantoea* and other associated microbes to assess their benefit on the host’s fitness. The reasons and factors causing the large size dispersion of associated bacterial populations and the relative abundance of *Pantoea* also warrant further study. The large size dispersion may have been caused by the timing and contents of feeding (Luo *et al.*, 2021). Differences in nutrition in the environments inhabited by insects may also affect population sizes and relative abundance. Further research efforts from the view of the associated microbiota are needed to obtain a more detailed understanding of the physiology and ecology of the sorghum plant bug and to achieve the control of this insect pest.

It is also important to note that *P. agglomerans* and *P. ananatis*, which formed the monophyletic clade with the OTUs and isolates derived from the sorghum plant bug, have been identified as rice pathogens (Cother *et al.*, 2004; Lee *et al.*, 2010). Although we need to carefully examine their pathogenicity, this finding indicates that these rice pathogens are brought into rice fields via the sorghum plant bug. The migration of plant pathogenic *Pantoea* spp. for‍ ‍other crops via various insects has been reported (Wielkopolan *et al.*, 2021; Coolen *et al.*, 2022). These findings suggest that the sorghum plant bug needs to be regarded and controlled not only as a notorious pest, but also as a potential vector of rice pathogenic *Pantoea* spp. In *P. agglomerans*, a lifestyle transition from a commensal to plant pathogenic style occurs via the acquisition of a plasmid-borne pathogenicity island (Barash and Manulis-Sasson, 2007). A transition between plant pathogenic and insect mutualistic lifestyles has also been reported in *Burkholderia* associated with *Largia* beetles (Flórez *et al.*, 2017). Lifestyle transitions may happen in the future or even now. Therefore, we will continue to examine the relationship between the sorghum plant bug and its associated *Pantoea* spp.

## Accession numbers

The nucleotide sequences of the 16S rRNA genes identified in the present study have been deposited in the DDBJ/EMBL/GenBank nucleotide sequence database under accession numbers LC743641 to LC743732 and LC757513 to LC757519. The sequence reads of amplicon sequencing have been deposited in the DDBJ Sequence Read Archive under accession numbers DRR425141 to DRR425169 (also see Supplementary Table S2 and S4 for details).

## Citation

Sato, Y., Akao, T., and Takeshita, K. (2023) High Prevalence of *Pantoea* spp. in Microbiota Associated with the Sorghum Plant Bug *Stenotus rubrovittatus* (Heteroptera: Miridae). *Microbes Environ ***38**: ME22110.

https://doi.org/10.1264/jsme2.ME22110

## Supplementary Material

Supplementary Material

## Figures and Tables

**Fig. 1.**
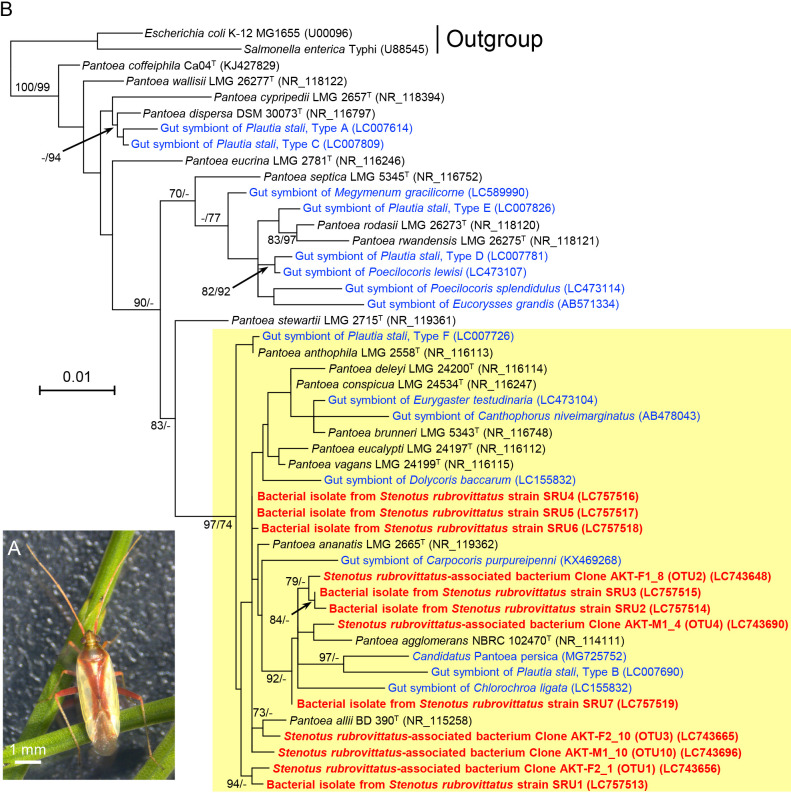
Maximum likelihood phylogeny of *Pantoea* clones/isolates derived from *Stenotus rubrovittatus* based on the 16S rRNA gene. (A) *S. rubrovittatus*. (B) The maximum likelihood phylogeny of the five *Pantoea* OTUs associated with *S. rubrovittatus*, the seven *Pantoea* isolates from *S. rubrovittatus*, and related *Pantoea* species/clones. The multiple alignments of the 1,338 nucleotide sites were analyzed. Phylogenetic relationships based on the maximum likelihood method were reconstructed with RAxML v8.2.12 using the general time reversible model with gamma distribution (GTR+Γ) (Stamatakis, 2014). The bootstrap values of 1,000 replicates were calculated with a rapid bootstrapping algorithm (Stamatakis *et al.*, 2008). A phylogenetic ana­lysis based on the neighbor joining method was also performed with MEGAX (Stecher *et al.*, 2020). Accession numbers in the DDBJ/EMBL/GenBank DNA database are shown in brackets. The *Pantoea* OTUs/isolates associated with *S. rubrovittatus* and *Pantoea* symbionts associated with other insects are shown in red and blue, respectively. The monophyletic clade containing the five *Pantoea* OTUs and seven *Pantoea* isolates is highlighted in yellow. Bootstrap support values higher than 70% are shown on the internal branches in the order of the maximum likelihood/neighbor joining method.

**Fig. 2.**
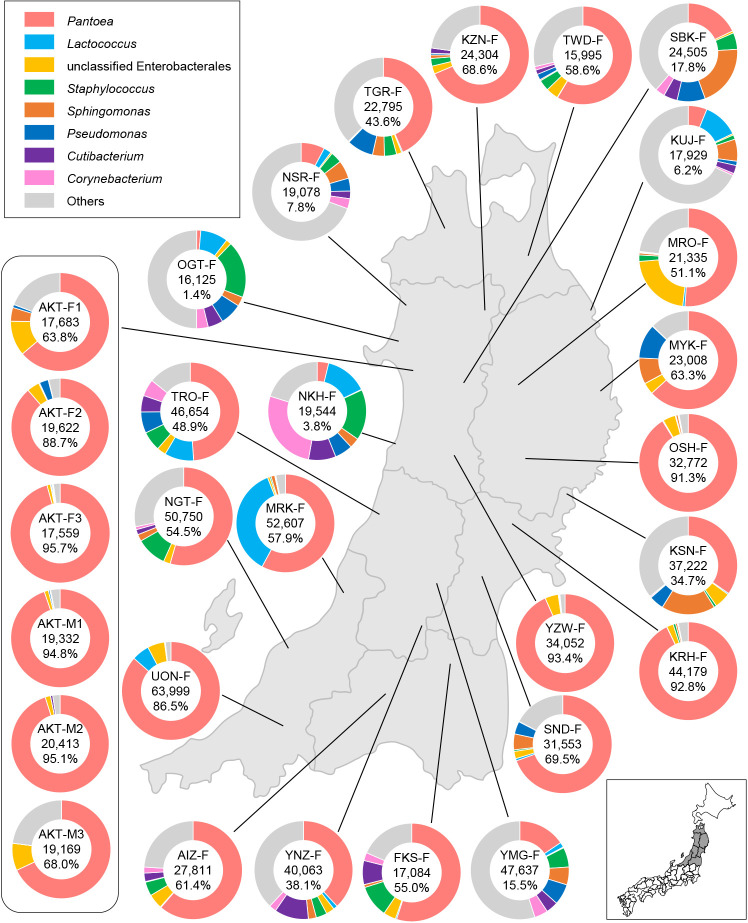
Relative abundance of the *Stenotus rubrovittatus*-associated microbiota at the genus level. The inset under the right shows a map of Japan, except for its small islands. Twenty-nine insect samples were collected at 24 locations across seven prefectures in the northern part of Honshu Island in Japan, which are indicated in gray in the map. The pie chart shows the relative abundance of the eight major taxa estimated by 16S rRNA amplicon sequencing. The sample name, number of sequences analyzed after removing Chloroplast sequences, and the percentage of *Pantoea* are shown in the center of the pie chart.

## References

[B1] Barash, I., and Manulis-Sasson, S. (2007) Virulence mechanisms and host specificity of gall-forming *Pantoea agglomerans*. Trends Microbiol 15: 538–545. 18024130 10.1016/j.tim.2007.10.009

[B2] Bourtzis, K., and Miller, T.A. (eds). (2003) *Insect Symbiosis*. Boca Raton, FL: CRC Press.

[B3] Buchner, P. (1965) *Endosymbiosis of Animals with Plant Microorganisms*. New York, NY: Interscience Publishers.

[B4] Chang, K.P., and Musgrave, A.J. (1970) Ultrastructure of rickettsia-like microorganisms in the midgut of a plant bug, *Stenotus binotatus* Jak. (Heteroptera: Miridae). Can J Microbiol 16: 621–622. 4097197 10.1139/m70-104

[B5] Coolen, S., Rogowska-van der Molen, M., and Welte, C.U. (2022) The secret life of insect-associated microbes and how they shape insect–plant interactions. FEMS Microbiol Ecol 98: fiac083. 35830517 10.1093/femsec/fiac083 PMC9409087

[B6] Cother, E.J., Reinke, R., McKenzie, C., Lanoiselet, V.M., and Noble, D.H. (2004) An unusual stem necrosis of rice caused by *Pantoea ananas* and the first record of this pathogen on rice in Australia. Australas Plant Pathol 33: 495–503.

[B7] Dally, M., Lalzar, M., Belausov, E., Gottlieb, Y., Coll, M., and Zchori-Fein, E. (2020) Cellular localization of two *Rickettsia* symbionts in the digestive system and within the ovaries of the mirid bug, *Macrolophous pygmaeus*. Insects 11: 1–15. 10.3390/insects11080530 PMC7469188 32823761

[B8] Douglas, A.E. (2015) Multiorganismal insects: diversity and function of resident microorganisms. Annu Rev Entomol 60: 17–34. 25341109 10.1146/annurev-ento-010814-020822 PMC4465791

[B9] Duron, O., and Noël, V. (2016) A wide diversity of *Pantoea* lineages are engaged in mutualistic symbiosis and cospeciation processes with stinkbugs. Environ Microbiol Rep 8: 715–727. 27362408 10.1111/1758-2229.12432

[B10] Flórez, L.V., Scherlach, K., Gaube, P., Ross, C., Sitte, E., Hermes, C., et al. (2017) Antibiotic-producing symbionts dynamically transition between plant pathogenicity and insect-defensive mutualism. Nat Commun 8: 15172. 28452358 10.1038/ncomms15172 PMC5414355

[B11] Hosokawa, T., Kikuchi, Y., Shimada, M., and Fukatsu, T. (2007) Obligate symbiont involved in pest status of host insect. Proc R Soc B 274: 1979–1984. 10.1098/rspb.2007.0620 PMC2275188 17567556

[B12] Hosokawa, T., Ishii, Y., Nikoh, N., Fujie, M., Satoh, N., and Fukatsu, T. (2016) Obligate bacterial mutualists evolving from environmental bacteria in natural insect populations. Nat Microbiol 1: 15011. 27571756 10.1038/nmicrobiol.2015.11

[B13] Hosokawa, T., Imanishi, M., Koga, R., and Fukatsu, T. (2019) Diversity and evolution of bacterial symbionts in the gut symbiotic organ of jewel stinkbugs (Hemiptera: Scutelleridae). Appl Entomol Zool 54: 359–367.

[B14] Itoh, H., Matsuura, Y., Hosokawa, T., Fukatsu, T., and Kikuchi, Y. (2017) Obligate gut symbiotic association in the sloe bug *Dolycoris baccarum* (Hemiptera: Pentatomidae). Appl Entomol Zool 52: 51–59.

[B15] Itoh, H., Tago, K., Hayatsu, M., and Kikuchi, Y. (2018) Detoxifying symbiosis: microbe-mediated detoxification of phytotoxins and pesticides in insects. Nat Prod Rep 35: 434–454. 29644346 10.1039/c7np00051k

[B16] Lee, H.B., Hong, J.P., and Kim, S.B. (2010) First report of leaf blight caused by *Pantoea agglomerans* on rice in Korea. Plant Dis 94: 1372. 10.1094/PDIS-05-10-0374 30743637

[B17] Luo, J., Cheng, Y., Guo, L., Wang, A., Lu, M., and Xu, L. (2021) Variation of gut microbiota caused by an imbalance diet is detrimental to bugs’ survival. Sci Total Environ 771: 144880. 33736123 10.1016/j.scitotenv.2020.144880

[B18] Lv, L., Luo, J., Ahmed, T., Zaki, H.E.M., Tian, Y., Shahid, M.S., et al. (2022) Beneficial effect and potential risk of *Pantoea* on rice production. Plants 11: 2608. 36235474 10.3390/plants11192608 PMC9570785

[B19] Osawa, T., Yamasaki, K., Tabuchi, K., Yoshioka, A., Ishigooka, Y., Sudo, S., and Takada, M.B. (2018) Climate-mediated population dynamics enhance distribution range expansion in a rice pest insect. Basic Appl Ecol 30: 41–51.

[B20] Rognes, T., Flouri, T., Nichols, B., Quince, C., and Mahé, F. (2016) VSEARCH: a versatile open source tool for metagenomics. PeerJ 4: e2584. 27781170 10.7717/peerj.2584 PMC5075697

[B21] Sato, Y., Jang, S., Takeshita, K., Itoh, H., Koike, H., Tago, K., et al. (2021) Insecticide resistance by a host-symbiont reciprocal detoxification. Nat Commun 12: 6432. 34741016 10.1038/s41467-021-26649-2 PMC8571283

[B22] Stamatakis, A., Hoover, P., and Rougemont, J. (2008) A rapid bootstrap algorithm for the RAxML web servers. Syst Biol 57: 758–771. 18853362 10.1080/10635150802429642

[B23] Stamatakis, A. (2014) RAxML version 8: a tool for phylogenetic ana­lysis and post-ana­lysis of large phylogenies. Bioinformatics 30: 1312–1313. 24451623 10.1093/bioinformatics/btu033 PMC3998144

[B24] Stecher, G., Tamura, K., and Kumar, S. (2020) Molecular evolutionary genetics ana­lysis (MEGA) for macOS. Mol Biol Evol 37: 1237–1239. 31904846 10.1093/molbev/msz312 PMC7086165

[B25] Stoddard, S.F., Smith, B.J., Hein, R., Roller, B.R.K., and Schmidt, T.M. (2015) rrnDB: improved tools for interpreting rRNA gene abundance in bacteria and archaea and a new foundation for future development. Nucleic Acids Res 43: D593–D598. 25414355 10.1093/nar/gku1201 PMC4383981

[B26] Tabuchi, K., Ichita, T., Ohtomo, R., Kashin, J., Takagi, T., Niiyama, T., et al. (2015) Rice bugs in the Tohoku region: their occurrence and damage from 2003 to 2013. Bull Tohoku Agric Res Cent 117: 63–115.

[B27] Walterson, A.M., and Stavrinides, J. (2015) *Pantoea*: insights into a highly versatile and diverse genus within the Enterobacteriaceae. FEMS Microbiol Rev 39: 968–984. 26109597 10.1093/femsre/fuv027

[B28] Wang, Q., Garrity, G.M., Tiedje, J.M., and Cole, J.R. (2007) Naïve Bayesian classifier for rapid assignment of rRNA sequences into the new bacterial taxonomy. Appl Environ Microbiol 73: 5261–5267. 17586664 10.1128/AEM.00062-07 PMC1950982

[B29] Wielkopolan, B., Jakubowska, M., and Obrępalska-Stęplowska, A. (2021) Beetles as plant pathogen vectors. Front Plant Sci 12: 748093. 34721475 10.3389/fpls.2021.748093 PMC8549695

[B30] Xue, H., Zhu, X., Wang, L., Zhang, K., Li, D., Ji, J., et al. (2021) Gut bacterial diversity in different life cycle stages of *Adelphocoris suturalis* (Hemiptera: Miridae). Front Microbiol 12: 670383. 34149656 10.3389/fmicb.2021.670383 PMC8208491

[B31] Yanai, T., and Iga, T. (1956) Further study on the binucleated epithelial cells in the mid-intestine of Heteropterous insects. Cytologia 21: 183–187.

[B32] Yasunaga, T., Takai, M., and Kawasawa, T. (eds) (2001) *A Field Guide to Japanese Bugs II*. Tokyo: Zenkoku Noson Kyoiku Kyokai (in Japanese).

