# Supplementary material for: High Prevalence of Pantoea spp. in Microbiota Associated with the Sorghum Plant Bug Stenotus rubrovittatus (Heteroptera: Miridae)
Source: Microbes Environ. 2023 Jul 13;38(3):ME22110. doi: 10.1264/jsme2.ME22110 (PMC10522847; doi:10.1264/jsme2.ME22110)
Supplement: Supplementary file 1 — Supplementary Material [file 38_22110_s1.pdf]

Supplementary file of:

Investigation of microbiota associated with an insect pest of rice *Stenotus rubrovittatus* (Heteroptera: Miridae).

Yuto Sato[1], Tatsuki Akao[1], Kazutaka Takeshita[1]*

[1]Faculty of Bioresource Sciences, Akita Prefectural University, 010-0195 Akita City, Japan

*Corresponding author: Kazutaka Takeshita

E-mail: kazu-t@akita-pu.ac.jp

This file includes:

Supplementary Materials and Methods.

Supplementary Fig. S1. Digestive tract of an adult female of *S. rubrovittatus*.

Supplementary Table S1. Primers used in the present study.

Supplementary Table S2. Insect samples and summary statistics of 16S rRNA amplicon sequencing.

Supplementary Table S3. Numbers of 16S rRNA gene clones assigned in each OTU.

Supplementary Table S4. List of cultured isolates from *S. rubrovittatus*.

Supplementary Table S5. Number of sequences detected in the *S. rubrovittatus*-associated microbiota at the genus level.

Supplementary Materials and Methods

Insect samples: Sampling was performed at 24 locations over seven prefectures (29 samples in total) in the northern part of Honshu Island in Japan (Supplementary Table S2). Adult insects were collected by sweeping for weeds in rice fields with an insect net. They were preserved in acetone until use (Fukatsu, 1999).

DNA extraction: DNA extraction from the whole insect body was performed with Wizard Genomic DNA Purification Kit (Promega), according to the manufacturer's instructions. The success of DNA extraction was confirmed by measuring DNA concentration with QuatiFluor ONE dsDNA System (Promega) and performing PCR amplification of the insect cytochrome oxidase 1 gene with TaKaRa EX Taq Hot Start Version (Takara Bio) (Supplementary Table S1 and Table S2). The composition of the PCR reaction mixture (total 10 µl) was as follow: 1 µl of 10×Ex Taq Buffer (Mg2+ plus) (20 mM), 0.8 µl of dNTP Mixture (2.5 mM each), 0.4 µl of primer mixture (10 µM each of forward and reverse primers), 1 µl of DNA sample, 0.05 µl of TaKaRa Ex Taq HS (5 U/µl), and 6.75 µl of distilled water. The temperature profile for the PCR was as follows:

94˚C for 2 min; and 30 cycles of 98˚C for 10 sec, 45˚C for 30 sec, and 72˚C for 1 min.

Clone library analysis: For the clone library analysis, a 1.5-kb fragment of bacterial 16S rRNA gene was amplified with TaKaRa EX Taq Hot Start Version (Supplementary Table S1). The composition of the PCR reaction mixture (total 10 µl) was as follow: 1 µl of 10×Ex Taq Buffer (Mg^{2+} plus) (20 mM), 0.8 µl of dNTP Mixture (2.5 mM each), 0.4 µl of primer mixture (10 µM each of forward and reverse primers), 1 µl of DNA sample, 0.05 µl of TaKaRa Ex Taq HS (5 U/µl), and 6.75 µl of distilled water. The temperature profile for the PCR was as follows: 94˚C for 2 min; 35 cycles of 98˚C for 10 sec, 52˚C for 30 sec, and 72˚C for 2 min; and 72˚C for 10 min. Cloning of the PCR products, Sanger sequencing of the clones, and assembling of the sequences were performed as described previously (Takeshita *et al.*, 2015). Clone sequences were classified into operational taxonomic units (OTUs) with vsearch 2.15.0 with a 99% identity threshold (Rognes *et al.*, 2016). The representative sequences of each OTU were subjected to a BLASTN search (Camacho *et al.*, 2009) against the NCBI 16S ribosomal RNA BLAST database (downloaded in March 2021).

Culture of the associated bacteria: Two adult females collected in the Akita campus, Akita prefectural university, Japan in Sep. 2020, were surface-sterilized by sinking in 70% ethanol for about 1 min, washed with phosphate-buffered saline (PBS: 137 mM NaCl, 2.7 mM KCl, 8.1 mM Na_2HPO_4, 1.5 mM KH_2PO_4 [pH 7.4]) adequately, and dissected in PBS with micro-scissors and micro-tweezers under a dissection microscope (S9 D, Leica Microsystems). The dissected digestive tract was photographed with a digital microscope camera (MC170 HD, Leica Microsystems). Each component of the digestive tract was separately homogenized in PBS, and then the diluted homogenates were plated on R2A (R2A broth "DAIGO", FUJIFILM Wako Pure Chemical Corp.) -agar and LB (1% Tryptone, 1% NaCl, 0.5% yeast extract) -agar plates. These plates were incubated at room temperature for 1-2 days. Emerging yellowish colonies, which is a characteristic of *Patnoea* spp. (Walterson and Stavrinides, 2015), were randomly picked up, and cultured in R2A or LB media at room temperature for 1 day. Stocks of them were stored in 20% glycerol at -80˚C. The 16S rRNA sequences of the isolates were determined and their homology search was performed, as described above.

Phylogenetic analysis: The sequences of the representative from each of the five *Pantoea* OTU, the seven *Pantoea* isolates, those of the related *Pantoea* symbionts from other insects, and those of type strains of the representative *Pantoea* spp., as well as outgroup sequences, were subjected to phylogenetic analysis based on the maximum likelihood method. Multiple alignment was constructed by using SINA v1.7.2 (Pruesse *et al.*, 2012), and gap-including and ambiguous sites in the alignment were then removed. Phylogenetic relationships based on maximum likelihood method were reconstructed with RAxML v8.2.12 using the general time reversible model with gamma distribution (GTR+Γ) (Stamatakis, 2014). The bootstrap values of 1,000 replicates were calculated with a rapid bootstrapping algorithm (Stamatakis *et al.*, 2008). Phylogenetic reconstruction and bootstrap analysis based on neighbor joining method were performed with MEGAX using the Tamura-Nei model with gamma distribution (TN+Γ) (Stecher *et al.*, 2020).

Quantitative PCR: For estimating the total number of bacteria associated with the insect host, qPCR targeting a 300-bp fragment of the bacterial 16S rRNA gene was performed

(Supplementary Table S1). The composition of qPCR reaction mixture (total 20 µl) was as follow: 10 µl of Luna Universal qPCR Master Mix (New England Biolabs), 4 µl of DNA sample, 1 µl of primer mixture (10 µM each of forward and reverse primers), and 5 µl of distilled water. The temperature profile for qPCR was as follows: 95˚C for 2 min; and 40 cycles of 95˚C for 15 sec, 54˚C for 10 sec, and 60˚C for 30sec. For calculating the absolute copy number, 10-fold serial dilutions of the target PCR product of *Escherichia coli* DH5α were applied.

Amplicon sequencing: PCR amplification of V3-V4 region of the bacterial 16S rRNA gene for amplicon sequencing was performed with TaKaRa EX Taq Hot Start Version (Supplementary Table S1). The composition of the PCR reaction mixture (total 30 µl) was as follow: 3 µl of 10×Ex Taq Buffer (Mg2+ plus) (20 mM), 2.4 µl of dNTP Mixture (2.5 mM each), 3 µl of primer mixture (10 µM each of forward and reverse primers), 1 µl of DNA sample, 0.3 µl of TaKaRa Ex Taq HS (5 U/µl), and 20.3 µl of distilled water. The temperature profile for the PCR was as follows: 94˚C for 2 min; 35 cycles of 94˚C for 30 sec, 55˚C for 30 sec, and 72˚C for 30 sec; and 72˚C for 5 min. Library preparation

and amplicon sequencing with Illumina MiSeq platform (2×300 bp) were conducted at Bioengineering Lab. Co., Ltd.

Adapter sequencing were removed from raw sequence reads with Cutadapt Version 3.4 (Martin, 2011). Merging the paired reads and quality filtering was performed with fastp 0.20.1 (Chen *et al.*, 2018). Low-quality reads ($Q < 30$, length < 100 bp) and short merged sequences (length < 400 bp) were removed before and after merging, respectively. Chimeric sequences were removed with chimera.uchime of mothur v.1.40.4 (Schloss *et al.*, 2009). The resulting sequences were taxonomically assigned at the genus level with RDP Classifier 2.13 with 80% confidence threshold (Wang *et al.*, 2007). The sequences assigned into Chloroplast were removed from the subsequent analyses.

References

Camacho, C., Coulouris, G., Avagyan, V., Ma, N., Papadopoulos, J., Bealer, K., and Madden, T.L. (2009) BLAST+: architecture and applications. *BMC Bioinformatics* **10**: 421.

Chen, S., Zhou, Y., Chen, Y., and Gu, J. (2018) fastp: an ultra-fast all-in-one FASTQ preprocessor. *Bioinformatics* **34**: i884–i890.

Fukatsu, T. (1999) Acetone preservation: a practical technique for molecular analysis. *Mol Ecol* **8**: 1935–1945.

Martin, M. (2011) Cutadapt removes adapter sequences from high-throughput sequencing reads. *EMBnet.journal* **17**: 10.

Pruesse, E., Peplies, J., and Glöckner, F.O. (2012) SINA: accurate high-throughput multiple sequence alignment of ribosomal RNA genes. *Bioinformatics* **28**: 1823–1829.

Rognes, T., Flouri, T., Nichols, B., Quince, C., and Mahé, F. (2016) VSEARCH: a versatile open source tool for metagenomics. *PeerJ* **4**: e2584.

Schloss, P.D., Westcott, S.L., Ryabin, T., Hall, J.R., Hartmann, M., Hollister, E.B., et al. (2009) Introducing mothur: open-source, platform-independent, community-supported software for describing and comparing microbial communities. *Appl Environ Microbiol* **75**: 7537–7541.

Stamatakis, A. (2014) RAxML version 8: a tool for phylogenetic analysis and post-analysis of large phylogenies. *Bioinformatics* **30**: 1312–1313.

Stamatakis, A., Hoover, P., and Rougemont, J. (2008) A rapid bootstrap algorithm for the RAxML web servers. *Syst Biol* **57**: 758–771.

Stecher, G., Tamura, K., and Kumar, S. (2020) Molecular evolutionary genetics analysis (MEGA) for macOS. *Mol Biol Evol* **37**: 1237–1239.

Takeshita, K., Matsuura, Y., Itoh, H., Navarro, R., Hori, T., Sone, T., et al. (2015) *Burkholderia* of plant-beneficial group are symbiotically associated with bordered plant bugs (Heteroptera: Pyrrhocoroidea: Largidae). *Microbes Environ* **30**: 321–329.

Walterson, A.M. and Stavrinides, J. (2015) *Pantoea*: insights into a highly versatile and diverse genus within the Enterobacteriaceae. *FEMS Microbiol Rev* **39**: 968–984.

Wang, Q., Garrity, G.M., Tiedje, J.M., and Cole, J.R. (2007) Naïve Bayesian classifier

for rapid assignment of rRNA sequences into the new bacterial taxonomy. *Appl Environ Microbiol* **73**: 5261–5267.

Supplementary Fig. S1.

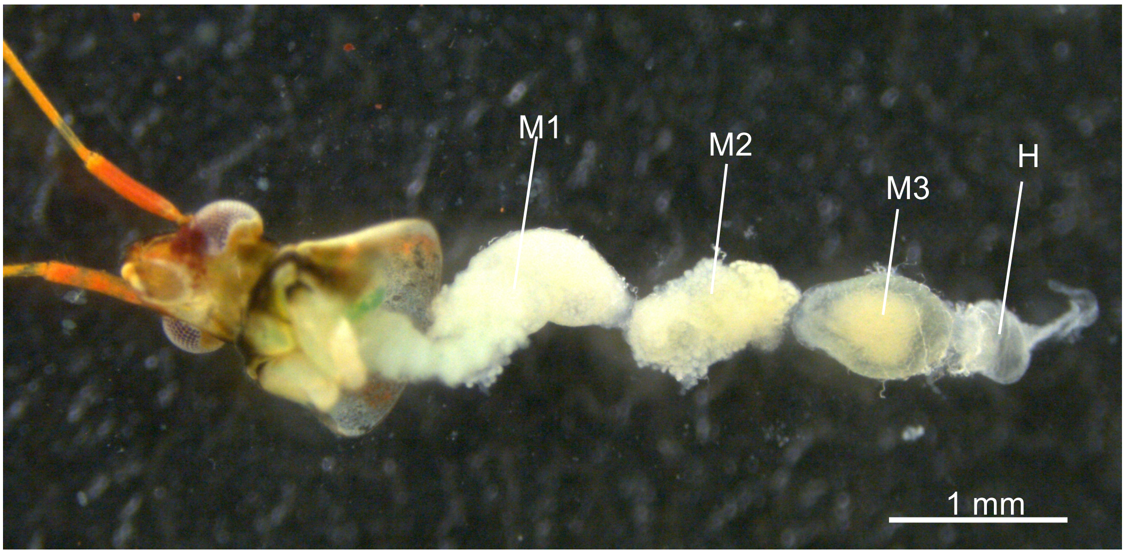

Supplementary Fig. S1. Digestive tract of an adult female of *S. rubrovittatus*. M1: midgut first section; M2: midgut second section; M3: midgut third section; and H: hindgut

Supplementary Table S1. Primers used in this study.

Purpose	Target	Name	Sequence (5' to 3')	Reference
Quality check	cytochrome oxidase 1 of invertebrates	LCO1490	GGTCAACAAATCATAAAGATATTGG	Folmer et al., 1994
		HCI2198	TAAACTTCAGGGTGACCAAAAAATCA	Folmer et al., 1994
Clone library analysis	16S rRNA of bacteria	16SA1 (27F)	AGAGTTTGATCMTGGCTCAG	Fukatsu & Nikoh, 1998 (Weisburg et al., 1991)
		16SB1	TACGGYTACCTTGTTACGACTT	Fukatsu & Nikoh, 1998
Sequencing	Universal	M13 Forward (-20)	GTAAAACGACGGCCAG	Thermo Fisher Scientific Inc. #N52002
		M13 Reverse	CAGGAAACAGCTATGAC	Thermo Fisher Scientific Inc. #N53002
	16S rRNA of bacteria	515F	GTGCCAGCMGCCGCGGTAA	Caporaso et al., 2012
		Eub920r	CCGYCAATTCCTTTGAGTTT	Shinzato et al., 2007
Amplicon sequencing	16S rRNA of bacteria	341F	CCTACGGGNGGCWGCAG[a]	Illumina Inc.
		805R	GACTACHVGGGTATCTAATCC[a]	Illumina Inc.
qPCR	16S rRNA of bacteria	515F	GTGCCAGCMGCCGCGGTAA	Caporaso et al., 2012
		806R	GGACTACHVGGGTWTCTAAT	Caporaso et al., 2012

[a] Adapter and random sequences are not shown.

References:

Folmer, O., Black M., Hoeh W., Lutz, R., and Vrijenhoek R. 1994. DNA primers for amplification of mitochondrial cytochrome c oxidase subunit I from diverse metazoan invertebrates. Mol Mar Biol Biotechnol 3: 294–299.

Fukatsu, T., and Nikoh N. 1998. Two intracellular symbiotic bacteria from the mulberry psyllid Anomoneura mori (insecta Homoptera). Appl Environ Microbiol 64: 3599–3606.

Weisburg, W.G., Barns, S.M., Pelletier, D.A., and Lane D.J. 1991. 16S ribosomal DNA amplification for phylogenetic study. J bacteriol 173(2): 697–703.

Caporaso, J.G., Lauber C.L., Walters W.A., Berg-Lyons D., Huntley J., Fierer N., et al. 2012. Ultra-high-throughput microbial community analysis on the Illumina HiSeq and MiSeq platforms. ISME J 6: 1621–1624.

Shinzato, N., Watanabe I., Meng X.Y., Sekiguchi Y., Tamaki H., Matsui T, et al. 2007. Phylogenetic analysis and fluorescence in situ hybridization detection of archaeal and bacterial endosymbionts in the anaerobic ciliate Trimyema compressum. Microb Ecol 54: 627–636.

Illumina Inc., 16S Metagenomic Sequencing Library Preparation, Part #15044223 Rev.B

Supplementary Table S2. Insect samples and summary statistics of the 16S rRNA amplicon sequencing.

Sample Name	Sex	Location	Sampling Date	DNA amount (μg)	Population Size (16S rRNA gene copies/ insect)[b]	Accession No.[c]	Accession No.[d]	No. of Sequences[e] Qualified	Bacteria[f]	*Pantoea*	Relative Abundance of *Pantoea* [e]
AKT-F1[a]	Female	Akita City, Akita	2019-08-27	1.5	5.507E+05	LC743641-LC743655	DRR425141	17,709	17,683	11,281	63.8%
AKT-F2[a]	Female	Akita City, Akita	2019-08-27	1.4	4.889E+05	LC743656-LC743671	DRR425142	20,169	19,776	17,395	88.0%
AKT-F3[a]	Female	Akita City, Akita	2019-08-27	1.6	9.688E+05	LC743672-LC743686	DRR425143	17,885	17,652	16,811	95.2%
AKT-M1[a]	Male	Akita City, Akita	2019-08-27	0.11	4.092E+05	LC743687-LC743702	DRR425144	20,166	20,039	18,327	91.5%
AKT-M2[a]	Male	Akita City, Akita	2019-08-27	0.14	1.354E+05	LC743703-LC743717	DRR425145	20,413	20,413	19,408	95.1%
AKT-M3[a]	Male	Akita City, Akita	2019-08-27	0.16	5.203E+05	LC743718-LC743732	DRR425146	19,443	19,348	13,044	67.4%
OGT-F	Female	Ogata, Akita	2019-09-21	3.8	3.479E+04	-	DRR425147	16,501	16,195	220	1.4%
NSR-F	Female	Noshiro, Akita	2019-08-27	1.4	4.289E+03	-	DRR425148	19,080	19,080	1,484	7.8%
KZN-F	Female	Kazuno, Akita	2019-08-27	0.58	2.010E+05	-	DRR425149	26,777	26,215	16,679	63.6%
SBK-F	Female	Senboku, Akita	2019-10-05	1.7	1.003E+04	-	DRR425150	25,314	24,769	4,374	17.7%
NKH-F	Female	Nikaho, Akita	2019-09-15	3.9	2.904E+03	-	DRR425151	19,545	19,544	738	3.8%
YZW-F	Female	Yuzawa, Akita	2019-09-15	4.5	2.761E+06	-	DRR425152	34,127	34,121	31,809	93.2%
TGR-F	Female	Tsugaru, Aomori	2020-09-11	1.6	2.621E+05	-	DRR425153	23,573	23,029	9,933	43.1%
TWD-F	Female	Towada, Aomori	2020-09-11	2.4	5.442E+05	-	DRR425154	16,668	16,385	9,371	57.2%
KUJ-F	Female	Kuji, Iwate	2020-09-11	2.6	1.248E+04	-	DRR425155	18,668	18,445	1,108	6.0%
MRO-F	Female	Morioka, Iwate	2020-09-09	1.4	4.365E+05	-	DRR425156	21,579	21,406	10,900	50.9%
MYK-F	Female	Miyako, Iwate	2020-09-11	5.4	2.035E+06	-	DRR425157	23,149	23,111	14,563	63.0%
OSH-F	Female	Oshu, Iwate	2020-09-09	4.6	1.204E+06	-	DRR425158	34,259	34,082	29,907	87.8%
KSN-F	Female	Kesennuma, Miyagi	2020-09-09	5.7	3.979E+04	-	DRR425159	38,926	38,586	12,913	33.5%
KRH-F	Female	Kurihara, Miyagi	2020-09-09	1.9	2.773E+06	-	DRR425160	44,541	44,468	40,998	92.2%
SND-F	Female	Sendai, Miyagi	2020-09-09	3.4	4.785E+05	-	DRR425161	34,097	33,535	21,921	65.4%
FKS-F	Female	Fukushima City, Fukushima	2020-08-23	3.6	2.275E+05	-	DRR425162	17,958	17,627	9,392	53.3%
AIZ-F	Female	Aizuwakamatu, Fukushima	2020-08-23	2.1	1.543E+06	-	DRR425163	28,391	28,165	17,068	60.6%
YNZ-F	Female	Yonezawa, Yamagata	2020-08-23	1.9	6.366E+03	-	DRR425164	43,094	41,731	15,267	36.6%
YMG-F	Female	Yamagata City, Yamagata	2020-08-23	0.76	1.021E+04	-	DRR425165	56,649	54,999	7,397	13.4%
TRO-F	Female	Tsuruoka, Yamagata	2020-08-26	0.96	1.605E+04	-	DRR425166	51,887	46,847	22,814	48.7%
MRK-F	Female	Murakami, Niigata	2020-08-26	2.4	1.981E+05	-	DRR425167	53,715	52,985	30,447	57.5%
NGT-F	Female	Niigata City, Niigata	2020-08-22	0.67	6.071E+04	-	DRR425168	55,266	54,346	27,634	50.8%
UON-F	Female	Uonuma, Niigata	2020-08-22	1.7	8.024E+06	-	DRR425169	63,999	63,999	55,375	86.5%

[a] The samples were also used for clone library analysis.

[b] Number of 16S rRNA gene copies per insect determined by qPCR is shown.

[c] Accession numbers of nucleotide sequences determined in the clone library analysis.

[d] Accession numbers of raw reads produced in the 16S rRNA amplicon sequencing.

[e] Summary statistics on the 16S rRNA amplicon sequencing.

[f] Sequences not assigned as bacteria were assigned as Eukaryota or not done into any domains (unclassified).

Supplementary Table S3. Numbers of 16S rRNA gene clones assigned in each OTU.

	Sample Name								Top hit[b]		
	AKT-F1	AKT-F2	AKT-F3	AKT-M1	AKT-M2	AKT-M3	Total	Accession No.[a]	Name	Accession No.	Identity (%)
OTU 1	3	6	7	13	2	7	38	LC743656	*Pantoea ananatis* LMG 2665	NR_119362	98.91
OTU 2	7		4		7	3	21	LC743648	*Pantoea agglomerans* NBRC 102470	NR_114111	99.04
OTU 3	2	5	3	1	2	2	15	LC743665	*Pantoea allii* BD 390	NR_115258	99.39
OTU 4		3		1	1		5	LC743690	*Pantoea ananatis* LMG 2665	NR_119362	98.74
OTU 5	2					3	5	LC743720	*Enterobacter ludwigii* EN-119	NR_042349	98.84
OTU 6		2					2	LC743663	*Pseudomonas oryzihabitans* NBRC 102199	NR_114041	99.72
OTU 7					2		2	LC743712	*Tundrisphaera lichenicola* P12	NR_158050	94.72
OTU 8	1						1	LC743655	*Sphingomonas sanguinis* NBRC 13937	NR_113637	99.13
OTU 9			1				1	LC743686	*Staphylococcus capitis* JCM 2420	NR_113348	99.73
OTU 10				1			1	LC743696	*Pantoea allii* BD 390	NR_115258	98.88
OTU 11					1		1	LC743710	*Cutibacterium acnes* JCM 6425	NR_113028	99.93
Total	15	16	15	16	15	15	92				

[a] Accession number of the representative clone sequence of the OTU.

[b] The result of BLASTN search against the NCBI 16S rRNA BLAST database.

Supplementary Table S4. The list of cultured isolates from *S. rubrovittatus*.

ID	Isolation source	medium	Accession No.	Top hit[a]		
				Name	Accession No.	Identity (%)
SRU1[b]	M1	LB	LC757513	*Pantoea ananatis* LMG 2665[e]	NR_119362	99.18
SRU2[b]	M2	LB	LC757514	*Pantoea agglomerans* NBRC 102470	NR_114111	98.91
SRU3[b]	M3	R2A	LC757515	*Pantoea ananatis* ATCC 33244[e]	NR_026045	99.17
SRU4[c,d]	H	LB	LC757516	*Pantoea allii* BD 390	NR_115258	99.32
SRU5[c,d]	M2	R2A	LC757517	*Pantoea allii* BD 390	NR_115258	99.32
SRU6[c]	M3	R2A	LC757518	*Pantoea allii* BD 390	NR_115258	99.39
SRU7[c]	H	R2A	LC757519	*Pantoea ananatis* LMG 2665[e]	NR_119362	99.04

[a] The result of BLASTN search against the NCBI 16S rRNA BLAST database.

[b,c] Letters indicate the isolates from the same individual.

[d] These sequences show 100% identity of each other.

[e] These strains are identical.

Supplementary Table S5. The number of detected sequences on *S. rubrovittatus* -associated microbiota at the genus level[a].

	Sample Name																												
Taxnomy	AKT-F1	AKT-F2	AKT-F3	AKT-M1	AKT-M2	AKT-M3	OGT-F	NSR-F	KZN-F	SBK-F	NKH-F	YZW-F	TGR-F	TWD-F	KUJ-F	MRO-F	MYK-F	OSH-F	KSN-F	KRH-F	SND-F	FKS-F	AIZ-F	YNZ-F	YMG-F	TRO-F	MRK-F	NGT-F	UON-F
Bacteria	17,683	19,776	17,652	20,039	20,413	19,348	16,195	19,078	26,215	24,769	19,544	34,121	23,029	16,385	18,445	21,406	23,111	34,082	38,586	44,468	33,535	17,627	28,165	41,731	54,999	46,847	52,985	54,346	63,999
Gammaproteobacteria/Enterobacterales/Erwiniaceae/Pantoea	11,281	17,395	16,811	18,327	19,408	13,044	220	1,484	16,679	4,374	738	31,809	9,933	9,371	1,108	10,900	14,563	29,907	12,913	40,998	21,921	9,392	17,068	15,267	7,397	22,814	30,447	27,634	55,375
Gammaproteobacteria/Enterobacterales/Erwiniaceae/unclassified_Erwiniaceae	176	580	275	528	337	197	7	21	600	63	25	420	156	178	7	4,073	226	343	211	1,173	664	175	336	284	121	364	288	655	789
Gammaproteobacteria/Enterobacterales/Enterobacteriaceae/Enterobacter	1,555	0	7	0	1	2,189	0	0	0	0	0	0	0	0	0	0	0	0	0	0	0	0	0	783	0	0	0	0	0
Gammaproteobacteria/Enterobacterales/Enterobacteriaceae/unclassified_Enterobacteriaceae	1,458	2	5	15	1	1,920	57	8	21	42	2	4	101	60	41	11	89	10	72	13	110	87	37	1,061	39	20	42	65	19
Gammaproteobacteria/Enterobacterales/unclassified_Enterobacterales	2,027	817	194	262	360	1,722	284	99	636	170	37	1,487	395	649	85	4,667	874	1,361	1,964	954	762	700	1,353	1,104	131	1,385	385	1,132	3,599
Gammaproteobacteria/Pseudomonadales/Pseudomonadaceae/Pseudomonas	176	580	61	93	0	0	1,165	809	138	2,263	1,150	0	1,982	307	253	44	2,607	25	1,796	134	1,309	3	6	0	3,262	3,258	8	0	0
Gammaproteobacteria/Pseudomonadales/Moraxellaceae/Acinetobacter	0	0	0	0	0	0	2,467	1,162	0	0	0	0	155	1,078	593	49	18	13	286	0	1,217	16	0	0	0	0	0	0	0
Gammaproteobacteria/Xanthomonadales/Xanthomonadaceae/Xanthomonas	0	0	0	0	0	0	29	6,306	0	0	0	0	118	34	3,033	0	42	0	931	0	0	19	0	0	0	0	0	0	0
Alphaproteobacteria/Sphingomonadales/Sphingomonadaceae/Sphingomonas	798	61	34	2	3	0	498	1,152	273	5,003	649	9	890	62	1,302	145	1,974	217	6,566	257	1,645	160	52	1,045	2,789	0	648	1,148	72
Alphaproteobacteria/Rhodobacterales/Rhodobacteraceae/Paracoccus	0	0	0	0	0	0	22	0	0	50	800	0	14	0	0	0	0	0	0	0	12	16	7	842	0	0	0	0	0
Alphaproteobacteria/Rhizobiales/Methylobacteriaceae/Methylobacterium	23	0	0	0	0	0	309	374	817	3,093	0	0	157	162	42	43	93	41	101	0	448	0	0	0	2,571	0	0	0	0
Alphaproteobacteria/Rhizobiales/Methylobacteriaceae/Methylorubrum	0	0	0	0	0	0	85	0	79	121	0	0	27	0	103	0	3	0	0	0	0	0	0	519	1,357	0	0	0	0
Alphaproteobacteria/Rhizobiales/Rhizobiaceae/Agrobacterium	0	0	0	0	0	0	71	0	0	533	0	0	90	42	658	86	61	0	747	0	0	15	0	0	900	0	0	0	0
Alphaproteobacteria/Rhizobiales/Aurantimonadaceae/Aureimonas	0	0	0	0	0	0	172	340	102	744	0	0	541	39	175	0	39	0	325	0	139	0	0	0	0	1,030	0	0	0
Betaproteobacteria/Burkholderiales/Burkholderiaceae/Burkholderia	0	0	0	0	0	0	661	0	0	0	1,140	0	74	853	2,516	46	0	0	0	0	0	0	0	0	0	0	0	0	0
Betaproteobacteria/Burkholderiales/Comamonadaceae/unclassified_Comamonadaceae	0	0	0	0	0	0	736	210	63	86	0	0	111	8	392	16	5	24	103	0	33	14	0	298	0	0	0	0	0
Betaproteobacteria/Neisseriales/Neisseriaceae/Neisseria	0	0	0	0	0	0	0	0	0	80	0	0	0	0	83	0	0	0	0	18	0	5	1	0	867	0	0	986	0
Betaproteobacteria/Neisseriales/Neisseriaceae/unclassified_Neisseriaceae	0	0	0	0	0	0	27	498	129	0	0	0	29	23	1	0	0	0	0	0	9	6	37	0	2,516	0	0	10	0
Bacilli/Lactobacillales/Streptococcaceae/Streptococcus	0	0	41	0	0	0	71	0	0	108	0	91	40	91	150	0	10	47	139	0	75	144	3,509	1,528	1,554	0	47	0	37
Bacilli/Lactobacillales/Streptococcaceae/Lactococcus	0	0	0	0	0	0	1,458	477	0	0	2,741	0	80	17	2,105	153	0	98	143	34	225	68	67	545	786	4,468	19,045	0	3,685
Bacilli/Bacillales/Staphylococcaceae/Staphylococcus	0	0	0	0	0	0	3,024	703	608	1,336	3,257	56	909	580	259	445	10	82	305	350	163	1,810	1,279	1,437	3,086	3,011	235	5,089	46
Negativicutes/Veillonellales/Veillonellaceae/Veillonella	0	0	0	0	0	0	89	0	0	0	0	0	0	86	0	0	0	0	0	0	0	0	1,118	0	494	0	61	0	0
Actinobacteria/Mycobacteriales/Corynebacteriaceae/Corynebacterium	0	0	31	47	0	0	616	627	0	769	5,278	52	40	183	130	30	13	30	140	0	14	451	550	945	2,105	2,600	115	485	20
Actinobacteria/Mycobacteriales/Lawsonellaceae/Lawsonella	0	0	0	0	0	0	712	393	54	109	0	26	27	137	66	76	8	0	30	64	66	1,393	309	855	791	1,620	51	404	39
Actinobacteria/Propionibacteriales/Propionibacteriaceae/Cutibacterium	0	31	46	0	144	1	802	474	421	1,106	1,757	61	36	239	485	37	22	67	55	8	24	1,351	770	4,482	1,903	2,517	68	848	41
Actinobacteria/Micrococcales/Microbacteriaceae/Curtobacterium	15	0	0	0	0	0	0	0	0	116	0	0	1,051	80	27	0	979	22	1,284	0	89	0	0	0	633	0	439	3,669	0
Actinobacteria/Micrococcales/Microbacteriaceae/Microbacterium	0	0	0	0	0	0	18	492	86	129	0	0	292	24	126	0	67	12	357	0	20	5	0	0	6,547	0	137	0	0
Actinobacteria/Micrococcales/Microbacteriaceae/unclassified_Microbacteriaceae	0	0	0	0	0	0	1	30	6	568	0	0	147	10	19	1	194	1	241	1	19	0	0	0	705	0	32	57	0
Flavobacteriia/Flavobacteriales/Weeksellaceae/Chryseobacterium	0	0	0	0	0	0	703	286	0	171	0	0	350	355	451	0	109	0	4,302	51	647	8	0	0	1,190	0	0	0	0
Sphingobacteriia/Sphingobacteriales/Sphingobacteriaceae/Mucilaginibacter	0	0	0	0	107	0	106	0	0	391	0	0	0	0	0	0	7	0	591	0	0	19	0	0	1,017	0	0	0	0
Cytophagia/Cytophagales/Hymenobacteraceae/Hymenobacter	0	0	0	0	0	0	0	0	718	679	0	0	744	95	367	0	24	0	324	0	501	37	0	0	0	0	0	0	0
Cytophagia/Cytophagales/Cytophagaceae/Spirosoma	0	0	0	0	0	0	0	0	0	544	0	0	73	47	190	0	22	38	35	0	129	0	0	0	0	0	0	0	0
Deinococci/Deinococcales/Deinococcaceae/Deinococcus	0	0	0	0	0	0	0	0	0	145	0	0	78	28	130	16	29	0	546	0	8	0	0	1,103	0	769	262	1,445	0
Chloroplast/Chloroplast/Chloroplast/Streptophyta[b]	0	154	93	707	0	179	70	0	1,911	264	0	69	234	390	516	71	103	1,310	1,364	289	2,002	543	354	1,668	7,362	193	378	3,596	0
Alphaproteobacteria/Rickettsiales/Rickettsiaceae/Rickettsia[c]	0	0	0	0	0	0	0	0	0	12	0	0	0	0	0	0	0	0	0	0	0	0	0	0	0	0	0	0	0
unclassified_Bacteria	10	8	9	13	30	0	26	4	1,567	40	247	8	96	44	104	17	57	136	67	13	70	130	65	179	134	510	95	530	95
Other bacteria[d]	164	148	45	45	22	96	1,689	3,129	1,307	1,660	1,723	29	4,059	1,113	2,928	480	863	298	2,648	111	1,214	1,060	1,247	7,786	4,742	2,288	202	6,593	182

[a] Major 34 genera of which relative abundance is > 1% in at least two samples, as well as *Rickettia* and "unclassified_Bacteria," are listed.

[b] This was non-target sequences, therefore we removed from Fig. 2.

[c] Rickettsia includes "unclassified_Rickettsiaceae" and "unclassified_Rickettsiales."

[d] The sum of the number of sequences of minor OTUs.
